# FPR2 promotes invasion and metastasis of gastric cancer cells and predicts the prognosis of patients

**DOI:** 10.1038/s41598-017-03368-7

**Published:** 2017-06-09

**Authors:** Xi-Lu Hou, Cheng-Dong Ji, Jun Tang, Yan-Xia Wang, Dong-Fang Xiang, Hai-Qing Li, Wei-Wei Liu, Jiao-Xue Wang, He-Zhong Yan, Yan Wang, Peng Zhang, You-Hong Cui, Ji-Ming Wang, Xiu-Wu Bian, Wei Liu

**Affiliations:** 1 Department of Gastroenterology, The 105th Hospital of People’s Liberation Army, Hefei, Anhui 230031 China; 2 Institute of Pathology & Southwest Cancer Center, and Key Laboratory of Tumor Immunopathology, Ministry of Education of China, Third Military Medical University, Chongqing, 400038 China; 3 Laboratory of Molecular Immunoregulation, Cancer and Inflammation Program, Center for Cancer Research, National Cancer Institute, Frederick, MD 21702 USA

## Abstract

Formyl peptide receptor 2 (FPR2), a classical chemoattractant receptor of G-protein-coupled receptors, is reported to be involved in invasion and metastasis of some cancers, but the role of FPR2 in gastric cancer (GC) has not yet been elucidated. In this study, we found that the levels of FPR2 expression in GC were positively correlated with invasion depth, lymph node metastasis and negatively correlated with the patients’ overall survival. Multivariate analysis indicated that FPR2 expression was an independent prognostic marker for GC patients. FPR2-knockdown significantly abrogated the migration and invasion stimulated by Hp(2–20) and Ac(2–26), two well-characterized ligands for FPR2 in GC cells. FPR2 deletion also reduced the tumorigenic and metastatic capabilities of GC cells *in vivo*. Mechanistically, stimulation with FPR2 ligands resulted in down-regulation of E-cadherin and up-regulation of vimentin, which were reversed by FPR2 knock-down, implying the involvement of epithelial–mesenchymal transition (EMT). Moreover, the activation of FPR2 was accompanied with ERK1/2 phosphorylation, which could be attenuated by FPR2 silencing or treatment with MEK inhibitor, PD98059. Altogether, our results demonstrate that FPR2 is functionally involved in invasion and metastasis, and potentially acts as a novel prognostic marker as well as a potential therapeutic target in human GC.

## Introduction

Gastric cancer (GC) is the fifth most common malignancy, and the third leading cause of cancer-related deaths worldwide^1^. Although remarkable achievements in surgical and other therapeutic options have been obtained, the overall 5-year survival rate of GC patients is still low^2^, mainly due to the advanced stage at diagnosis and the malignant nature of invasion and metastasis of the disease. Therefore, new insights into the mechanisms underlying the invasion and metastasis is crucial for development of novel agents to improve clinical outcome of GC patients.

Formyl peptide receptor 2 (FPR2) belongs to the 7 transmembrane G-protein–coupled FPR family that comprises another two members FPR1 and FPR3 in humans^3^. FPR2 was originally identified in phagocytic leukocytes and plays an important role in host defense by mediating leukocyte chemotaxis upon activation by bacterial and host-derived agonists^4–6^. Subsequently, FPR2 was reported to be also present in non-myeloid cells^7^, involving in colonicepithelial homeostasis^8^ and several human diseases, such as Alzheimer’s disease (AD)^9^ and prion disease^10^. Recently, experimental evidence suggests that FPR2 is associated with the cancers. FPR2 was expressed on ovarian cancer cells and required for LL-37-induced invasion of the cells^11^. FPR2 was also overexpressed in primary melanoma and correlated with aggressive tumor characteristics^12^. In colon cancer, elevated FPR2 expression was associated with poorer patient prognosis^13^. However, the potential function of FPR2 in GC is poorly understood.

FPR2 is a promiscuous receptor in response to a variety of structurally diverse ligands, such as fMLF, prion peptide (PrP106–126)^10^, lipoxin A4^14^, Hp(2–20)^15^, annexin A1 (ANXA1) and its N-terminal peptide Ac(2–26)^16^, and various synthetic peptides^6^. Among them, Hp(2–20) and Ac(2–26) are present in the gastric tissue and function in a ligand-specific manner^17–20^. Hp(2–20), an exogenous ligand of FPR2, is a cecropin-like peptide derived from *Helicobacter pylori*, which is a recognized risk factor of gastric cancer^21^. Hp(2–20) stimulation induced the migration and proliferation of GC cells by activating FPR2^18^. Ac(2–26), an endogenous ligand of FPR2 derived from ANXA1, has been found to activate FPR2 to enhance invasion of GC cells^22^. These reports suggest that FPR2 might play important roles in carcinogenesis and progress of GC.

In this study, we evaluated the relevance between the expression of FPR2 and the clinical characteristics in GC. The results indicated that FPR2 was overexpressed in GC tissues and was an independent prognostic factor for the patients. Mechanistically, we demonstrated that FPR2 could enhance capabilities of invasion and metastasis of GC cells by activating MAPK/ERK pathway to induce EMT.

## Results

### FPR2 expression is associated with clinicopathological characteristics and outcome of GC patients

**Figure 1:**
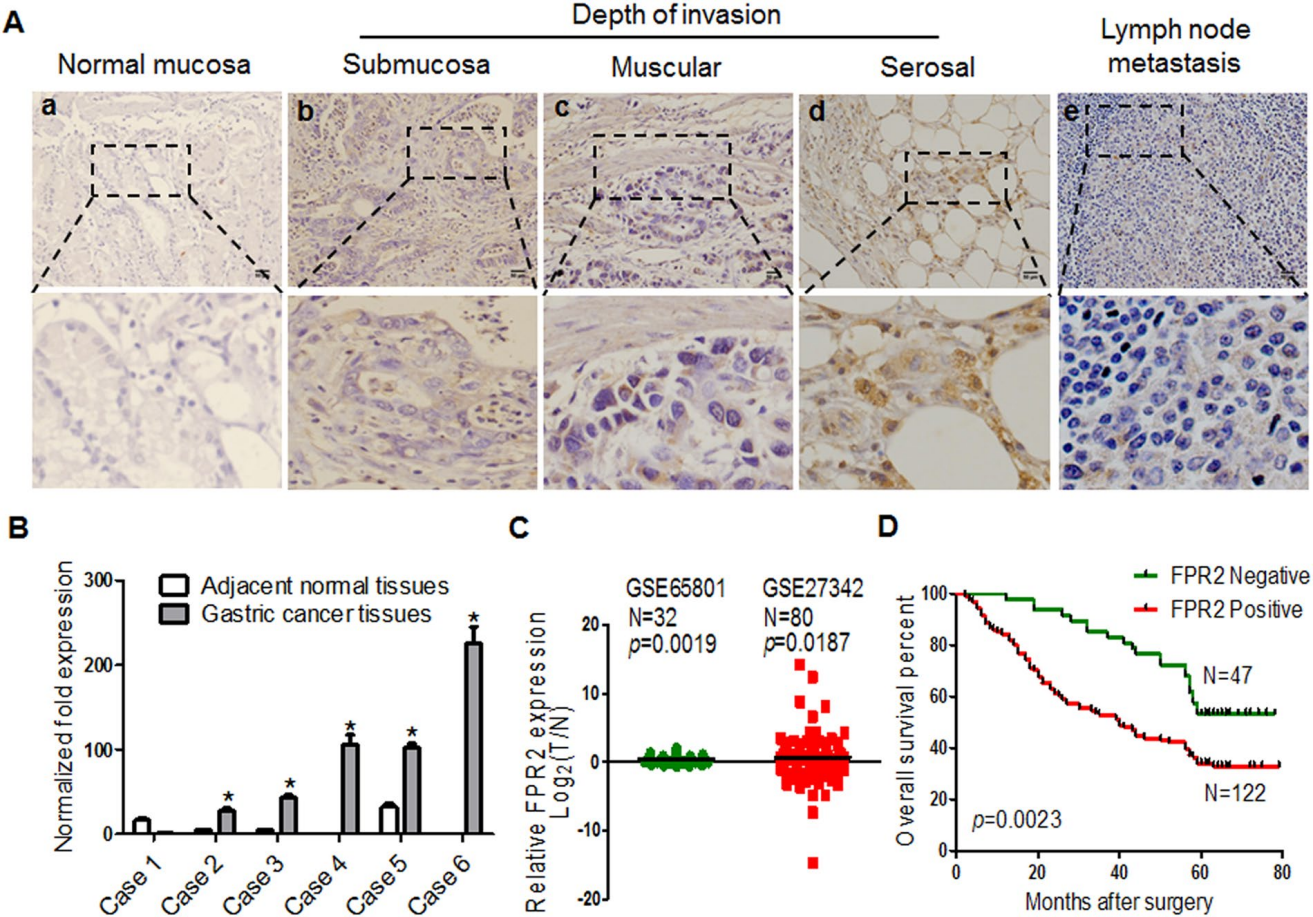
FPR2 expression in gastric cancer tissues is correlated with the clinicopathological characteristics and outcome of GC patients. (**A**) Representative images of FPR2 IHC staining in normal mucosa tissues, GC tissues and metastatic lymph node. (a) FPR2 is negatively or weakly expressed in normal stomach mucosa. (b–d) The levels of FPR2 expression are elevated with the invasion depth in GC tissues. (e) GC metastatic focus of lymph node showing FPR2 positive staining. (**B**) Quantitative analysis of FPR2 mRNA in 6 fresh surgical tumor specimens and paired adjacent normal tissues. (**C**) The mRNA expression of FPR2 in gastric cancer and paired normal adjacent tissues from 2 reported datasets. (**D**) Kaplan-Meier overall survival curves of 169 GC patients indicates that FPR2^+^ patients hold shorter life time -then FPR2^−^ patients (*p* = 0.0023). Scale bar = 50 μm.

To elucidate the clinical relevance of FPR2 in the human GC, immunohistochemistry (IHC) was performed to detect the levels of FPR2 expression in tumor tissues and their adjacent normal tissues from 169 GC patients. The FPR2 protein was mainly localized in the cytomembrane and cytoplasm of the cancer cells. The expression of FPR2 was very low or absent in normal gastric mucosa (Fig. 1Aa). The highly expressed FPR2 was observed in cancer tissues as well as in metastatic lymph nodes (Fig. 1Ab–e). As shown in Fig. 1Ab–d, the staining intensity of FPR2 increased with invasion depth. Among GC specimens, 122 (72.2%) showed positive expression (FPR2^+^) and 47 (27.8%) showed negative expression of FPR2 (FPR2^−^), while in corresponding adjacent normal tissues, 131 (77.5%) were FPR2^−^ and 38 (22.5%) appeared FPR2^+^ (*p* < 0.001, Table S1). In a separate set of samples, quantitative analysis of FPR2 mRNA in 6 fresh surgical specimens indicated that 5 out of 6 tumor tissues had high level of FPR2 expression as compared with their adjacent normal tissues (Fig. 1B). The expression profiling by array from 2 reported datasets (GSE65801 and GSE27342)^23, 24^ showed that the mRNA expression of FPR2 was significantly higher in gastric cancer tissues than in the paired normal adjacent tissues (Fig. 1C). The correlation analysis between FPR2 expression in cancerous tissues and clinicopathological features showed that FPR2+ was positively related to TNM (Tumor, Node, Metastasis) stage (*P* = 0.002), serosal invasion (*P* = 0.015) and lymph node metastasis (*P* = 0.043), but not with histological grade (*P* = 0.812, Table 1).

**Table 1:** The correlation between FPR2 expression and clinical pathologic parameters in GC.

Clinicopathological parameter	Total No.	FPR2		*p* value
		Negative (%)	Positive (%)	
Age, y				
≤60	108	34(31.5)	74(68.5)	0.156
>60	61	13(21.3)	48(78.7)	
Sex				
Female	51	22(43.2)	29(56.8)	0.003
Male	118	25(21.2)	93(78.8)	
Histological grade				
G1 + G2	49	13(26.5)	36(73.5)	0.812
G3	120	34(28.3)	86(71.7)	
TNM stage				
I	28	16(57.1)	12(42.9)	0.002
II	40	8(20)	32(80)	
III	67	17(25.4)	50(74.6)	
IV	34	6(17.6)	28(82.4)	
Serosal invasion				
Absent	52	21(40.4)	31(59.6)	0.015
Present	117	26(22.2)	91(77.8)	
Lymph node metastasis				
Absent	68	26(38.2)	42(61.8)	0.043
Present	101	24(23.8)	77(76.2)	

Kaplan-Meier analysis was performed to analyze the association of FPR2 expression with the overall survival rates of GC patients. The patients with FPR2^+^ had shorter lifespan compared to those with FPR2^−^ (*p* = 0.0023, Fig. 1D). Univariate and multivariate analyses showed that the expression of FPR2 was an independent prognostic indicator for the overall survival of GC patients (p = 0.002 and p = 0.026, respectively) (Table 2). To further reveal the prognostic significance of FPR2 expression in GC patients, Kaplan–Meier estimates were also performed in patients with different depth of invasion and with or without lymph node metastasis. In the serosal invasion group, patients with FPR2^+^ had worse overall survival compared to those with FPR2^−^ (*P* = 0.003, Fig. S1A), while similar lifespans of patients with FPR + or FPR^−^ were observed in non-serosal invasion patients group (*P* = 0.9702, Fig. S1B). In the group of lymph node metastasis, the overall survival of FPR2^+^ patients was worse than that of FPR2^−^ patients (*P* = 0.0145, Fig. S1C), but the overall survival of patients was not associated with FPR2 expression in the group of non-lymph node metastasis (*P* = 0.1909, Fig. S1D). These results suggest that FPR2 may exert important roles in carcinogenesis and progress of GC and serve as a prognostic biomarker for the patients.

**Table 2:** Univariate and Multivariate Analysis of the effect of FPR2 on survival.

	Univariate Analysis		Multivariate Analysis	
	Hazard Ratio(95%CI)	*p* value	Hazard Ratio(95%CI)	*p* value
Age	1.465(0.991–2.166)	0.056	1.235(0.812–1.879)	0.325
Sex	0.980(0.658–1.460)	0.922	0.968(0.632–1.481)	0.880
Histological grade	0.703(0.471–1.084)	0.084	0.753(0.491–1.156)	0.194
T stage	1.985(1.290–3.054)	0.002	1.259(0.724–2.187)	0.414
Lymph node metastasis	2.038(1.370–3.033)	0.000	1.000(0.557–1.795)	0.999
TNM	2.426(1.614–3.646)	0.000	2.175(1.130–4.187)	0.020
FPR2	1.145(1.053–1.246)	0.002	1.115(1.013–1.228)	0.026

### FPR2 promotes migration and invasion of GC cells *in vitro*

**Figure 2:**
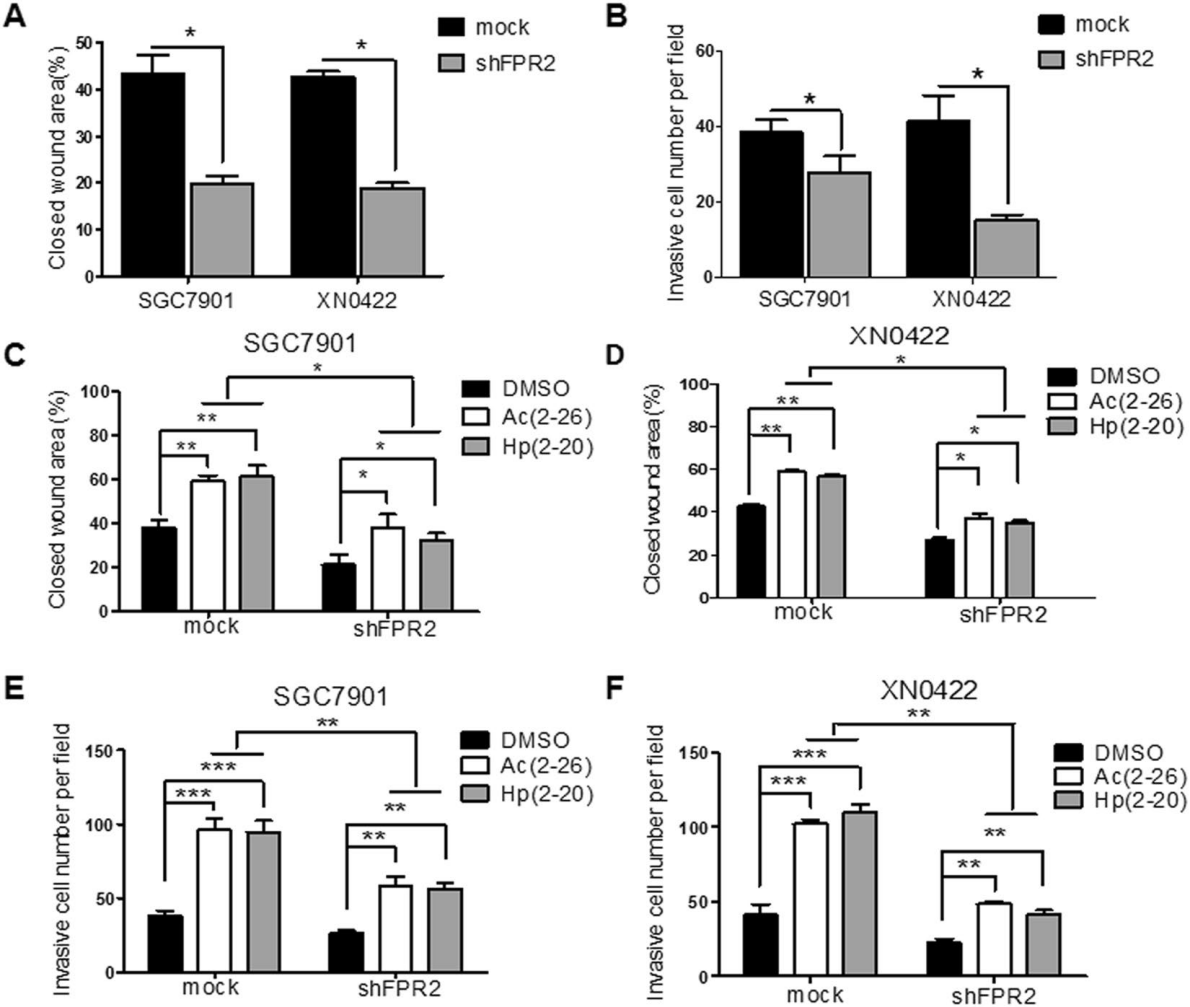
The migratory and invasive capabilities of GC cells are enhanced upon stimulation with FPR2 ligands and attenuated by FPR2-knockdown *in vitro*. (**A**) Wound healing experiments showed that shFPR2 SGC7901 and shFPR2 XN0422 cells migrate shorter distance than paired mock cells. (**B**) FPR2-knockdown impaired the invasive capabilities of SGC7901 and XN0422 cells. (**C** and **D**) FPR2 ligands Hp(2–20) and Ac(2–26) enhanced migratory capabilities of GC cells, which was attenuated by FPR2-knockdown. (**E** and **F**) FPR2 ligands enhanced invasive capabilities of GC cells, which was impaired by FPR2-knockdown. -Data are mean ± SD. *p *<* 0.05, **p *<* 0.01 and ***p *<* 0.001.

Since FPR2 expression was associated with invasion depth and lymph node metastasis in GC specimens, we then evaluated the abilities of migration and invasion of GC cells *in vitro*. To examine the effect of the FPR2 on migration and invasion, FPR2-knockdown primary GC cell XN0422 and GC cell line SGC7901 cells were treated with shRNA targeting FPR2 (Fig. S2). Wound healing assay showed that XN0422 and SGC7901 cells with FPR2 knockdown migrated into the scratching area more slowly than the mock cells (p < 0.05, Figs 2A and S3A). GC cells with FPR2-knockdown also exhibited decreased invasive capability (p < 0.05, Figs 2B and S3B). As shown in Figs 2C,D and S3C,D, stimulation with Hp(2–20) and Ac(2–26) promoted migration of both XN0422 and SGC7901 cells, and this effect was significantly impaired by FPR2-knockdown (p < 0.05 for all). Similarly, Hp(2–20) and Ac(2–26) enhanced the invasiveness of GC cells, which was markedly attenuated by deletion of FPR2 (Figs 2E,F and S3E,F). In addition, we noticed that FPR2-knockdown could only attenuate but not eliminate the effect of ligands (Figs 2C–F and S3C–F), the reason for which might be the redundant expressions of FPR1 and/or FPR3 in GC cells. In supporting out speculation, the different expression patterns of FPRs were observed in 6 GC cells (5 cell lines and a primary GC cell) and 6 fresh GC specimens, in which all 6 cells and specimens expressed FPRs, but the expression levels of FPR2 in 5 of 6 cells and specimens were much higher than that of FPR1 and FPR3 (Fig. S4A and B). On the other hand, Hp(2–20) and Ac(2–26) are not the specific ligands for FPR2, they can also interact with FPR1 and/or FPR3^6^. The results indicate that FPR2 can promote the migration and invasion of GC cells *in vitro*.

### FPR2 promotes tumorigenesis and metastasis of GC cells *in vivo*

**Figure 3:**
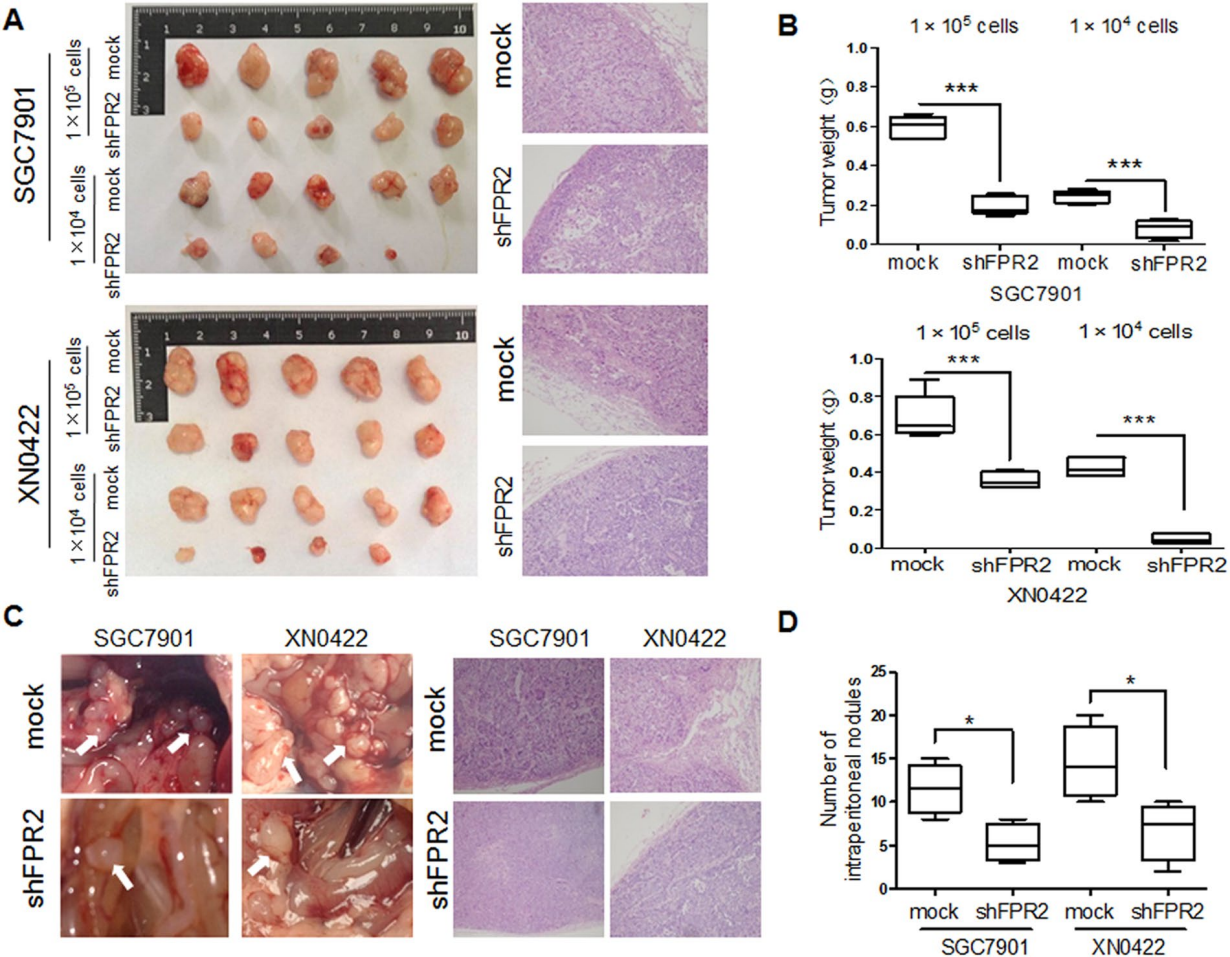
FPR2 promotes tumorigenesis and metastasis of GC cells *in vivo*. (**A**) Images of xenograft tumors derived from shFPR2 SGC7901 and shXN0422 and their mock cells subcutaneously injected in nude mice at indicated cell number for 5 weeks showed that FPR2-knockdown reduced the tumorigenic abilities of GC cells both in rate of tumor formation and tumor size (left panel). HE staining confirmed the GC pathological characteristics of xenograft tumors (right panel). (**B**) summary graphs showed the weights of tumors derived from FPR2-knockdown SGC7901 (upper) and XN0422 (lower) cells are significantly lighter than those derived from the mock cells. (**C**) Representative images of intraperitoneal metastasis assay showed that metastatic nodules derived from FPR2-knockdown GC cells are far less than those derived from the mock cells (left panel), and HE staining confirms the GC pathological characteristics of the metastatic nodules (right panel). (**D**) Histograms show the difference in number of celiac metastatic nodules derived from FPR2-knockdown GC cells and mock cells (each cell was implanted at 2 × 104 cells/mouse for 4 weeks, n = 5). *p < 0.05, ***p < 0.001.

Since FPR2 served as an inducer for migration and invasion of GC cells *in vitro*, we next examined the roles of FPR2 in tumorigenesis and metastasis of GC cells *in vivo*. Subcutaneous xenograft model was used to investigate the effect of FPR2 on tumorigenic ability in nude mice. Although both FPR2-knockdown shFPR2 SGC7901 or shFPR2 XN0422 cells and their mock cells at 1 × 10^5^ cells/mouse had capability to form xenograft tumors in all nude mice, the size and weight of tumors derived from FPR2-knockdown cells were markedly smaller and lighter than that of tumors formed by paired mock cells (Fig. 3A,B). When the implantation was performed at 1 × 10^4^ cells/mouse, not only the smaller and lighter xenograft tumors but also the reduced tumor-forming ratio (4/5 vs 5/5) were observed in FPR2-knockdown GC cells as compared to mock cells (Fig. 3A,B). An intraperitoneal metastasis model was employed to assess the role of FPR2 in metastasis of GC cells. shFPR2 SGC7901 and shFPR2 XN0422 and their mock cells were injected into the peritoneal cavity of nude mice at 2 × 10^4^ cells/mouse for 4 weeks, respectively (n = 5). Metastatic nodules were found in all the four groups, but SGC7901 and XN0422 mock cells generated more metastatic nodules compared with shFPR2 SGC7901 and shFPR2 XN0422 cells (11.50 ± 2.89 vs 5.25 ± 2.22 and 14.50 ± 4.20 vs 6.75 ± 3.40, respectively. p < 0.05) (Fig. 3C,D). These results strongly suggest that FPR2 plays important roles in tumorigenesis and metastasis of GC.

### The activation of FPR2 induces Epithelial-mesenchymal-transition in GC cells

**Figure 4:**
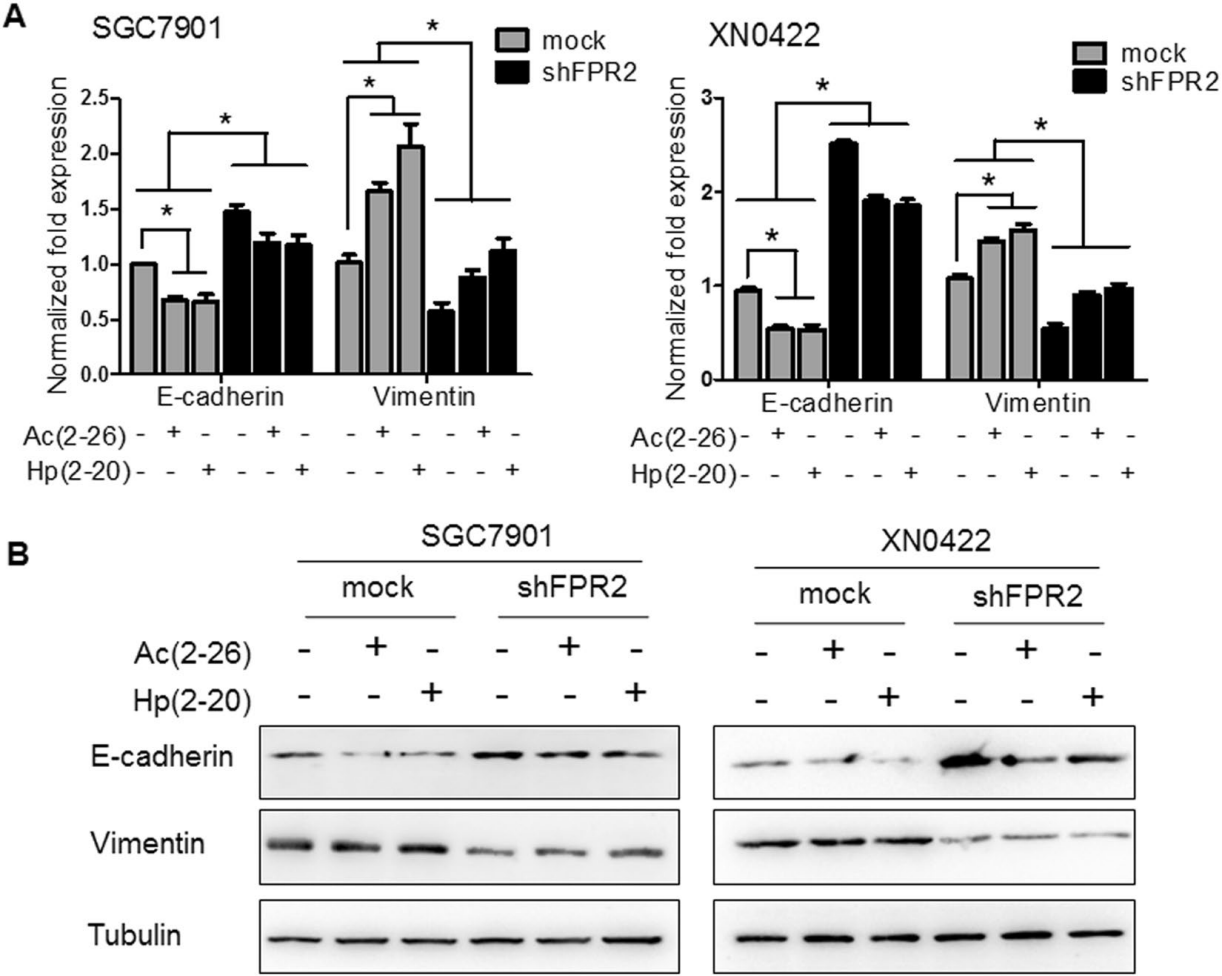
Activation of FPR2 is associated with the expression of EMT-related molecules in GC cells. (**A**) Stimulation with Hp(2–20) and Ac(2–26) (100 μM for each) down-regulated E-cadherin and up-regulateed vimentin expression in SGC7901 cells (left panel) and XN0422 cells (right panel) detected by real-time PCR and normalized against GAPDH, but these effects are reversed by FPR2-knockdown. (**B**) Western blot assay showed the same consequence as A at protein level.

Epithelial-mesenchymal-transition (EMT), which converts tumor cells into an elongated, motile and invasive phenotype, has been well recognized as pivotal incident for tumor cells to invasion and metastasis. To clarify whether EMT is involved in the FPR2-promoting invasion and metastasis, the expression of EMT-related molecules E-cadherin and vimentin was examined in GC cells with or without FPR2 knockdown under presence or absence of FPR2 ligands. As shown in Fig. 4A, treatment with FPR2 ligands Hp(2–20) and Ac(2–26) decreased mRNA expression of E-cadherin and enhanced mRNA expression of vimentin, while FPR2 knockdown reversed the expression patterns of these two molecules in SGC7901 cells (left panel) and XN0422 cells (right panel). Western blot assay confirmed these results at protein level (Fig. 4B). However, FPR2 knockdown could not abolish the effect of FPR2 ligands on the expression of EMT-related molecules, possibly due to the presence of FPR1 and FPR2 in GC cells and the non-specificity of the ligands. These results demonstrate that the activation of FPR2 can induce EMT of GC cells, which may be an important mechanism for FPR2-promoting invasion and metastasis of GC cells.

### FPR2-promoting migration involves the activation of ERK signaling pathway in GC cells

**Figure 5:**
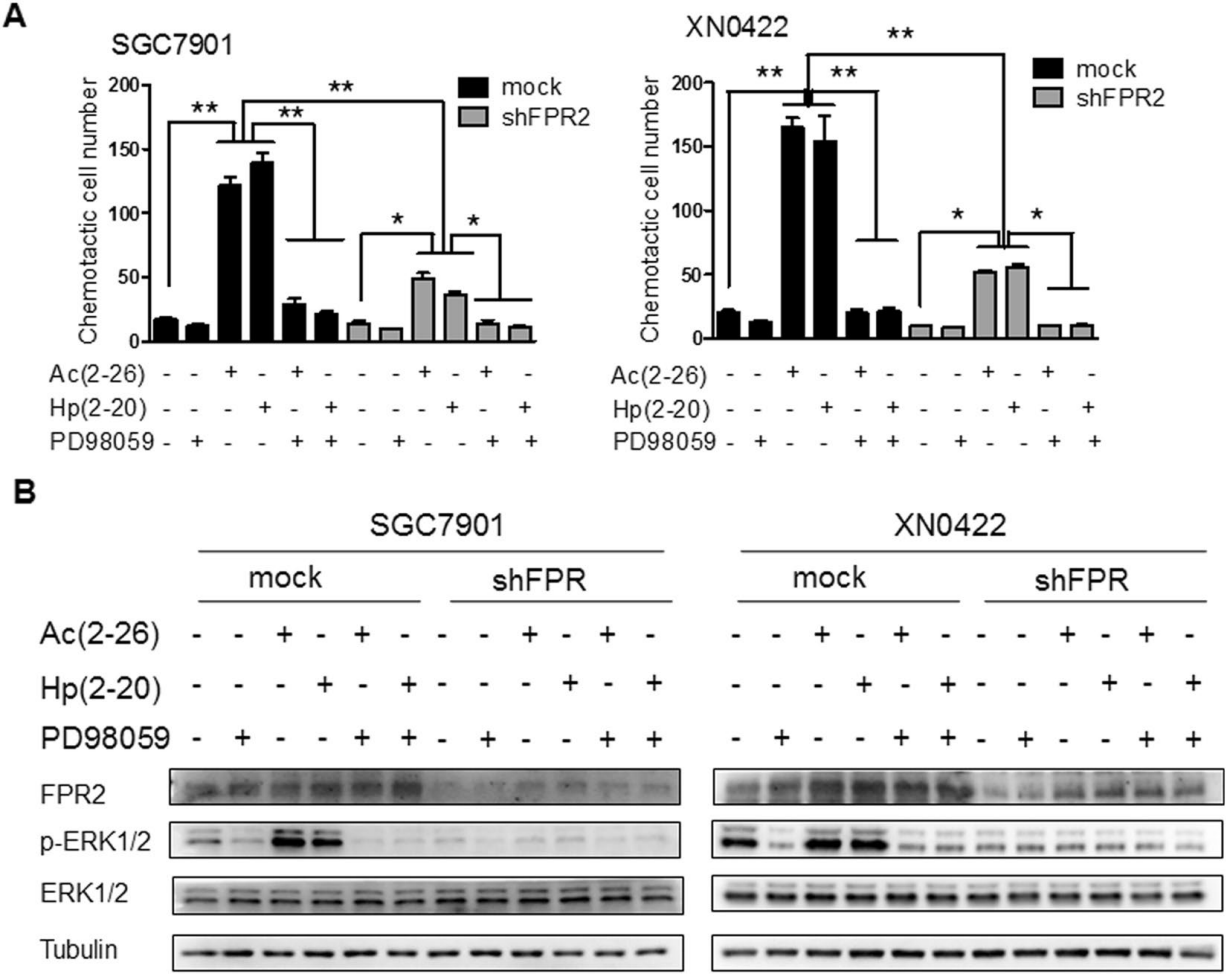
FPR2 induces migration of GC cells mainly by activating ERK signaling pathway. (**A**) Hp(2–20) and Ac(2–26) (100 μM) induces chemotaxis of SGC7901 cells (left panel) and XN0422 cells (right panel), which is markedly attenuated by FPR2 knockdown and blocked by treatment with MEK1/2 inhibitor PD98059 (10 μM). (**B**) Western blot showed that Hp(2–20) and Ac(2–26) (100 μM) induced ERK 1/2 phosphorylation in SGC7901 cells (left panel) and XN0422 cells (right panel), while FPR2 knockdown and PD98059 treatment inhibits ERK 1/2 phosphorylation. Data are presented as mean ± SD. *p < 0.05, **p < 0.01.

Activation of FPR2 leads to a series of signaling events, including phosphorylation of the protein kinase ERK1/2^25^. Upon activation, ERK1/2 activate multiple nuclear and cytoplasmic targets (>600), including EMT-related transcription factors and regulators of cell motility and invasion^26^. Therefore, we further investigated whether the activation of FPR2 evoked the activation of ERK signaling pathway. As shown in Fig. 5A, both Hp(2–20) and Ac(2–26) were able to induce chemotaxis in SGC7901 and XN0422 mock cells. These effects were nearly blocked by PD98059, a specific MEK inhibitor and markedly attenuated by FPR2 knockdown. ERK 1/2 phosphorylation was triggered by ligand treatment, but inhibited by PD98059 treatment and FPR2 knockdown both in SGC7901 and XN0422 cells (Fig. 5B). These results suggest that the activation of ERK signaling pathway is a critical event in FPR2-induced invasion and metastasis of GC cells.

## Discussion

All the members of FPR family, including FPR1, FPR2, and FPR3, are expressed in human GC cells^18^. The roles of FPR1 in GC have been contradictorily reported. Prevete *et al*.^27^ found that FPR1 is a tumor suppressor by inhibiting angiogenesis in GC xenograft experiments. Otani *et al*.^28^ reported that a specific FPR1 polymorphism, which reduced FPR1 activity, is positively associated with the risk of GC. While Cheng *et al*.^29^ reported that FPR1 was highly expressed in GC tissues and significantly associated with stage IV disease, invasion depth, and clinical outcome of the patients. So far, there is a few data to correlate the expression of FPR2 and FPR3 with GC. In this study, we first examined the expression patterns of FPRs in 6 GC cells and 6 fresh GC specimens and found that all GC cells and specimens expressed all the members of FPRs, but 5 of 6 GC cells and 5 of 6 GC specimens had much higher expression levels of FPR2 than FPR1 and FPR3. These prompted us to further investigate the role of FPR2 in GC. We found that FPR2 was expressed more frequently in GC cancerous tissues than in adjacent tissues and increased expression levels in cancerous tissues were correlated with the invasion depth and lymph node metastasis as well as the poor survival of the patients. To our knowledge, this is the first clinicopathological study to link FPR2 to the clinicopathological features of GC and the outcome of GC patients.

Invasion and metastasis are considered to be the main factors affecting the prognosis of patients with GC. Our clinical data suggested that FPR2 was a potential factor involved in facilitating invasion and metastasis of GC. It was confirmed by *in vitro* and *in vivo* experiments with GC cell line SGC7901 and primary GC cell XN0422. Silencing FPR2 expression significantly impaired the migratory and invasive potentials induced by Hp(2–20) and Ac(2–26) as well as the capability of peritoneal metastasis in the GC cells. Our *in vitro* experiments were in line with the report of Prevete *et al*.^27^, in which FPR2 promoting EMT and migration in GC cells, and another report, in which FPR2-promoting migration and invasion in human pancreatic carcinoma cells^30^. In our experiments, we also found that FPR2 deletion markedly decreased the tumor formation in nude mice. However, the findings of Prevete *et al*.^27^ showed that knockdown of FPR2 did not significantly affected GC cell tumor formation despite a significant decrease in cell growth *in vitro*.

EMT is a highly conserved and fundamental process that is critical for embryogenesis and tumor progression. More and more evidence suggests that the achievement of an EMT phenotype is associated with increased capability of invasion and metastasis in GC cells^31^. The up-regulation of mesenchymal markers and the down-regulation of epithelial markers are the major molecular features of EMT. Our and other’s^27^ works proved that FPR2 knockdown up-regulated mesenchymal marker vimentin and down-regulated epithelial maker E-cadherin in GC cells, suggesting that EMT is an important mechanism in FPR2-promoting GC invasion and metastasis.

FPR2 is a remarkably versatile receptor that can be activated by an array of ligands. Among them, Ac(2–26), an endogenous ligand derived from ANXA1, and Hp(2–20), an exogenous ligand derived from *Helicobacter pylori*, are well known to present in gastric tissue, but they are not specific for FPR2. The EC50 of Ac(2–26) for FPR1, FPR2 and FPR3 are 5 μM, 0.9 μM and >10 μM, respectively; while Hp(2–20) can only bind FPR2 and FPR3 with EC50 of 0.3 μM and 10 μM, respectively^5^, indicating that Ac(2–26) and Hp(2–20) have the greatest affinity for FPR2 compared with FPR1 and FPR3. Contradictory expression pattern and clinical relevance of ANXA1 have been reported in human gastric cancer. Cheng *et al*.^22^ reported that high AnxA1 expression was associated with more serosal invasion, more peritoneal metastasis, and poorer overall survival in GC patients. Sato *et al*.^32^ also reported that elevated ANXA1 expression was involved in GC invasion and lymph node metastasis and implicated in poor prognosis of the patients. ANXA1 was expressed both in cytoplasm and nucleus of GC cells. Zhu *et al*.^33^ found that the nuclear localization of ANXA1 correlated with advanced disease stage and peritoneal dissemination. However, Yu *et al*. reported that ANXA1 was a negative biomarker for gastric cancer development and progression^34^. Gao *et al*. also reported that ANXA1 was down-regulated in gastric cancer, and overexpression of ANXA1 in GC cells leads to cell growth inhibition^35^. In our study, we found that ANXA1-derived peptide Ac(2–26) could activate FPR2 to induce migration and invasion of GC cells. Although it is well-known that *Helicobacter pylori* infection is an important risk factor of GC, the clinical relevance of Hp(2–20) in GC has not been illustrated. However, it could regulate gastric mucosal healing by facilitating epithelial cell migration, proliferation and angiogenesis through interaction with FPR2 and FPR3^22^. In the present study, we found that Hp(2–20) could induce GC cell migration and invasion by activating FPR2, suggesting that Hp(2–20)/FPR2 interaction may be one of the mechanisms of *Helicobacter pylori* infection-induced GC progression.

Activation of FPR2 by binding with different ligands and in different cells triggers different signaling pathways, such as phospholipase C (PLC), protein kinase C (PKC) isoforms, phosphoinositide 3-kinase (PI3K)/protein kinase B (Akt), mitogen-activated protein kinase (MAPK), and so on^36^. The activation of MAPK/ERK pathway is a common event in tumorigenesis, and plays a critical role in cancer progression through regulating cell migration, apoptosis and proteinase induction^37^. Several studies have demonstrated that activation of FPR2 promoted tumor cell invasion by evoking MARK/ERK pathway^11, 38, 39^. In our study, ERK phosphorylation in GC cells could be excited by treatment with Ac(2–26) and Hp(2–20), while this response could be blocked by PD98059, a specific MEK inhibitor. These strongly suggested that FPR2 promotes GC progression mainly though activation of MAPK/ERK pathway.

In conclusion, we demonstrated that, for the first time, high expression of FPR2 in gastric cancer tissues is correlated with poor prognosis of GC patients. We also elucidated that FPR2 can enhance the invasion and metastasis of gastric cancer. A possible mechanism regarding these effects was that FPR2 promotes GC cell EMT by activating MAPK/ERK pathway. Thus, FPR2 could be potentially used as not only a prognostic biomarker but also a therapeutic target for GC patients. However, it is worth mentioning that the high FPR2 expression in gastric cancer might be a symptom of an underlying mechanism, which should be a target of therapeutic approaches besides FPR2 itself and its signaling pathway and needs to be further investigated.

## Material and Methods

### Patients and specimens

A total of 169 formalin-fixed and paraffin-embedded surgical carcinous and the corresponding adjacent normal tissues were collected from GC patients who were enrolled in the Southwest Hospital from January 2006 to December 2007. All patients had not received radiotherapy, chemotherapy or immunotherapy before surgery. Follow-up information was available for all patients for a period of minimum 80 months. All the specimens were routinely processed for pathological diagnosis according to the WHO classification. The study was approved by the Southwest Hospital Research Ethics Committees, and all patients were enrolled by written informed consent.

### Cells and culture

Human gastric cancer cell line SGC7901 was purchased from Cell Bank of Shanghai Institute of Cell Biology, Chinese Academy of Sciences and primary gastric cancer cell XN0422 was initiated by our laboratory. Both the cell line and primary cells were cultured in Roswell Park Memorial Institute 1640 (RPMI-1640) medium (Gibco, Grand island, USA) supplemented with 10% fetal bovine serum (BD Pharmingen, USA) in the condition of a humidified atmosphere containing 5% CO_2_ at 37 °C. Cells in exponential growth phase (approximately 80% confluency) were used in all experiments.

### Immunohistochemistry

After fixation in 4% formalin, cancerous and corresponding adjacent normal tissues from the 169 GC patients were dehydrated through an ascending series of graded ethanol, embedded in paraffin wax, and cut into 4-μm sections. After dewaxing and hydrating, antigen retrival, bloking of endogenous peroxidase activity, the sections were incubated with primary FPR2 antibody (1:100, Santa Cruz, USA) at 4 °C overnight. Following incubation with secondary antibody (Beijing Zhongshan Golden Bridge Biotechnology, China) at 37 °C for 30 minutes, the sections were visualized using diaminobenzidine solution (DAKO) and lightly counterstained with haematoxylin. The tumors were interpreted as FPR2-positive and FPR2-negative according to the cancer cells with or without staining of FPR2.

### RNA extraction and quantitative real-time PCR (qRT-PCR)

Total RNA was isolated using RNAiso TRIzol reagent (TAKARA, Kyoto, Japan) according to the manufacturer’s instructions. Reverse-transcription of RNA was performed in a final reaction volume of 20 μL containing 1000 ng of total RNA by using PrimeScript RT Master Mix (TAKARA, Kyoto, Japan). FPR2 mRNAs were detected by qRT-PCR with the SYBR Premix Ex TaqII (TAKARA, Kyoto, Japan). The sequences of all primers for RT-qPCR were presented in Table S2.

### Western blot analysis

The cells were lysed in RIPA Lysis Buffer (Beyotime Biotechnology, China) with 1 mM protease inhibitor PMSF (Thermo, USA). Protein concentration was determined using DAB (Thermo, USA). Then 30 μg of total protein was separated using 10% SDS-PAGE and transferred onto PVDF membranes (Thermo, USA). After blocked with 5% evaporated skimmed milk, the membranes were incubated with primary human FPR2 mouse mAb (1:500, Anogen, China), human E-cadherin mouse mAb (1:1000, Cell Signaling Technology, USA), human vimentin rabbit mAb (1:1000, Cell Signaling Technology, USA), ERK1/2 rabbit mAb (1:1000, Cell Signaling Technology, USA), phospho-ERK1/2 rabbit mAb (1:1000, Cell Signaling Technology, USA) and human Tubulin mouse mAb (1:1000, Beyotime Biotechnology, China) overnight at 4 °C, separately. After washing with PBST, membranes were incubated with appropriate HRP-conjugated secondary antibodies (1:5000) for 1.5 h at the room temperature, and then washed with PBST. Finally, the protein expression was visualized with an enhanced chemiluminescence (ECL) detection system (Thermo Scientific, Watertown, MA, USA). The protein gel images were acquired under automatic exposure settings on ChemiDoc™ MP System (Bio-Rad, Hercules, California, USA) with Image Lab (Version 5.2 build 14) software. Tubulin was used as a loading control.

### Lentivirus Production and Infection

Three shRNA sequences targeted against FPR2 and a non-targeting scrambled gene sequence were listed in Table S3. Lentivirus particles containing shFPR2 and control shRNA were obtained from Life Technologies Co. Ltd (Shanghai, China) and used to infect SGC7901 and XN0422 cells with 2 μg/mL of polybrene. The stable FPR2-knockdown cells were selected using 3 μg/mL puromycin. The efficacy of FPR2 knockdown at mRNA and protein levels were examined by RT-PCR and Western blot analysis, respectively (Fig. S2).

### Wound-healing assay

A wound-healing assay was performed to examine the capability of cancer cell migration, as previously described^40^. Briefly, GC cells were grown in 24-well plates with RPMI-1640 medium supplemented with 10% FBS up to 90% confluence. A single scratch wound was generated with a 10 μL pipette tip. After removing the suspension cells by washing with PBS, fresh RPMI-1640 medium without FBS was added. With a Live Cell Imaging System (ZEISS, Germany), moving and growing of cells across the scratched lines were monitored every hour for 24 h. The migratory ability of the cells was presented as the gap distance recovered compared with the original gap.

### Invasion assay *in vitro* and Chemotaxis assay

Invasion assay was performed as previously described^40^. Briefly, GC cells were seeded in the upper chamber (8 μm, 24-well format) coated with 10 μL of matrigel (BD, USA)/RPMI-1640 (1:1, v/v) at 2 × 104 cells/well in 200 μL RPMI-1640 medium without FBS. The lower chambers were filled with 600 μL RPMI-1640 medium containing 10% FBS. After 24 h of incubation, the membranes were fixed with 4% paraformaldehyde for 20 min. Then the non-invaded cells (upper surface of the membrane) were removed with a cotton swab, and the cells on the lower surface of the membrane were stained with crystal violet solution (Beyotime, China). The number of invaded cells was counted in five randomly selected high powered fields under a microscope. Chemotaxis assay was performed as previously described^41^. Briefly, transwell chambers (8 μm pore size, Millipore) without matrigel coating were used. The upper wells of the chamber were added with 5 × 10^4^ cells suspended in 200 μL serum-free RPMI-1640 medium. Lower wells of the chamber were added with 600 μL serum-free medium containing different concentrations of Hp(2–20) or Ac(2–26), which were synthesized by GL Biotech Ltd (Shanghai, China) according to their sequences (Table S3) and their biological activity was tested by chemotaxis (Fig. S5). After an incubation period of 6 h at 37 °C, migrated cells on the lower surface of membrane were counted in five randomly chosen fields.

### Subcutaneous xenograft tumorigenicity and intraperitoneal metastasis assays

To assess the effect of FPR2 on *in vivo* tumorigenecity, XN0422 and SGC7901 FPR2-knockdown and mock cells were injected subcutaneously into axilla of 6-week-old female nude mice (Laboratory Animal Center, Third Military Medical University) at 1 × 10^4^ cells and 1 × 10^5^ cells suspended in 0.2 mL Matrigel (1:1, v/v) per mouse, respectively (n = 5). The mice were euthanized at the end of 5 weeks after implantations. The xenografts were removed and measured. The tumorigenic capability was assessed by tumor weight. To examine the effect of FPR2 on *in vivo* metastasis, the GC cells with different treatments were injected intraperitoneally (1 × 10^4^ cells per mouse). After 4 weeks, the mice were euthanized. The numbers of intraperitoneal nodules were counted. All animal procedures were approved by the Third Military Medical University Animal Committee.

### Statistics

All data are expressed as mean ± SD of three independent experiments and analyzed using SPSS 18.0 statistical software. Kaplan-Meier analysis was used to evaluate the survival rates and chi-square test was used to detect the associations between FPR2 expression and clinicopathologic characteristics of GC patients. To determine whether FPR2 is an independent prognosis factor for survival, the Cox proportional hazards model was used to calculate the hazard ratios. The statistical significance of the mean values was evaluated using the unpaired Student’s t test. Tests were assumed significant when the P < 0.05.

All the methods were carried out in accordance with relevant approved guidelines and regulations.

## Electronic supplementary material

Supplementary Figures and Tables
