# Supplementary material for: FPR2 promotes invasion and metastasis of gastric cancer cells and predicts the prognosis of patients
Source: Sci Rep. 2017 Jun 9;7:3153. doi: 10.1038/s41598-017-03368-7 (PMC5466646; doi:10.1038/s41598-017-03368-7)
Supplement: Supplementary file 1 — Supplementary Figures and Tables [file 41598_2017_3368_MOESM1_ESM.pdf]

FPR2 promotes invasion and metastasis of gastric cancer cells and predicts the prognosis of patients

Xi-Lu Hou[1#], Cheng-Dong Ji[2#], Jun Tang[1#], Yan-Xia Wang[2], Dong-Fang Xiang[2], Hai-Qing Li[1], Wei Liu[1], Jiao-Xue Wang[1], He-Zhong Yan[1], Yan Wang[2], Peng Zhang[2], You-Hhong Cui[2], Ji Ming Wang[3], Xiu-Wu Bian[2*], Wei Liu[1*]

[1]Department of Gastroenterology, The 105th Hospital of People's Liberation Army, Hefei, Anhui 230031, China.

[2]Institute of Pathology & Southwest Cancer Center, and Key Laboratory of Tumor Immunopathology, Ministry of Education of China, Third Military Medical University, Chongqing 400038, China

[3]Laboratory of Molecular Immunoregulation, Cancer and Inflammation Program, Center for Cancer Research, National Cancer Institute, Frederick, MD 21702, USA.

Supplementary tables

Table S1. IHC staining of FPR2 in gastric cancer and paired adjacent tissues

	FPR2		*p* value
	Negative (%)	Positive (%)	
Gastric cancer tissue	47(27.8)	122(72.2)	<0.0001
Adjacent tissue	131(77.5)	38(22.5)	

Table S2. Sequences of primer for real-time PCR used in this study

Name	Sequence
GAPDH	F: 5’-AAGGTGAAGGTCGGAGTCAAC-3’<br>R: 5’-GGGGTCATTGATGGCAACAATA-3’
FPR2	F: 5’TTCACGGCCACATTACCATTC -3’<br>R: 5’-AATCCAAGGTCCGACGATCAC -3’
E-Cadherin	F: 5’-CCCACCACGTACAAGGGTC-3’<br>R: 5’-ATGCCATCGTTGTTCACTGGA-3’
Vimentin	F: 5’- GGGACCTCTACGAGGAGGAG -3’<br>R: 5’- CGCATTGTCAACATCCTGTC -3’

Table S3. Sequences of FPR2 shRNA and scrambled control

shRNA	Sequence (5' - 3')
#1	CCGGGGTGATCGTCGGACCTTGGATTCTTCTCGAGAAGAATCCAAGGTC CGACGATCACCTTTTTG
#2	CCGGGGATTATCCGGTTTGTCATTGGCTTCTCGAGAAGCCAATGACAAA CCGGATAATCCTTTTTG
#3	CCGGAAGTGTCTTCTTGATTGGTTTCATTCTCGAGAATGAAACCAATCA AGAAGACACTTTTTTTG
Scrambled control	CCGGTTACGCGTAGCGTAATACGCTCGAGCGTATTACGCTACGCGTAA TTTTTG

Table S4. Sequences of Ac(2-26) and Hp(2-20) peptides

Peptide	Sequence
Ac(2-26)	Ac-AMVSEFLKQAWFIENEEQEYVQTVK
Hp(2-20)	AKKVFKRLEKLFSKIQNDK

Supplementary Figures

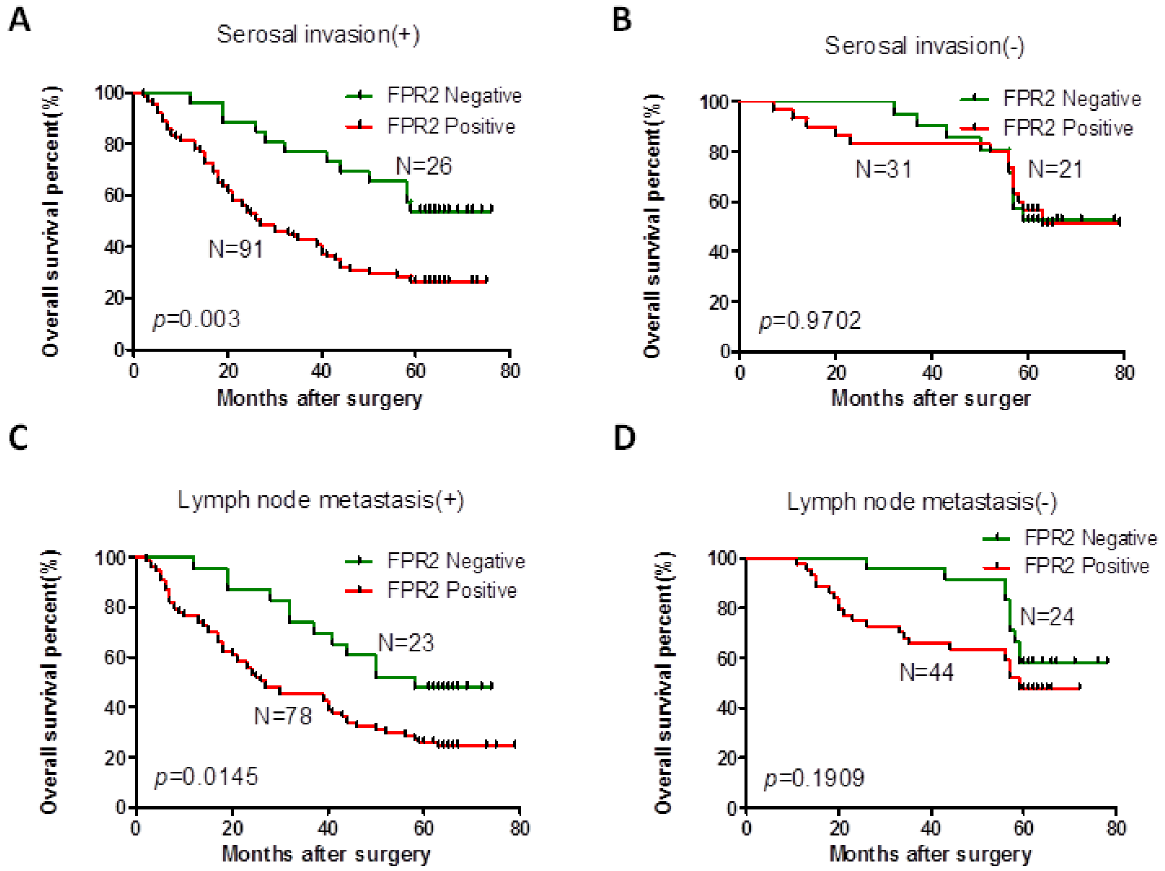

Figure S1. Kaplan–Meier analysis of correlation between FPR2 expression and overall survival rate in different groups of GC patients with different status of invasion and lymph node metastasis. A, Kaplan-Meier estimation of overall survival for serosal invasion patients. B, Kaplan-Meier estimation of overall survival for patients without serosal invasion. C, Kaplan-Meier estimation of overall survival for patients with lymph node metastasis. D, Kaplan-Meier estimation of overall survival for patients without lymph node metastasis.

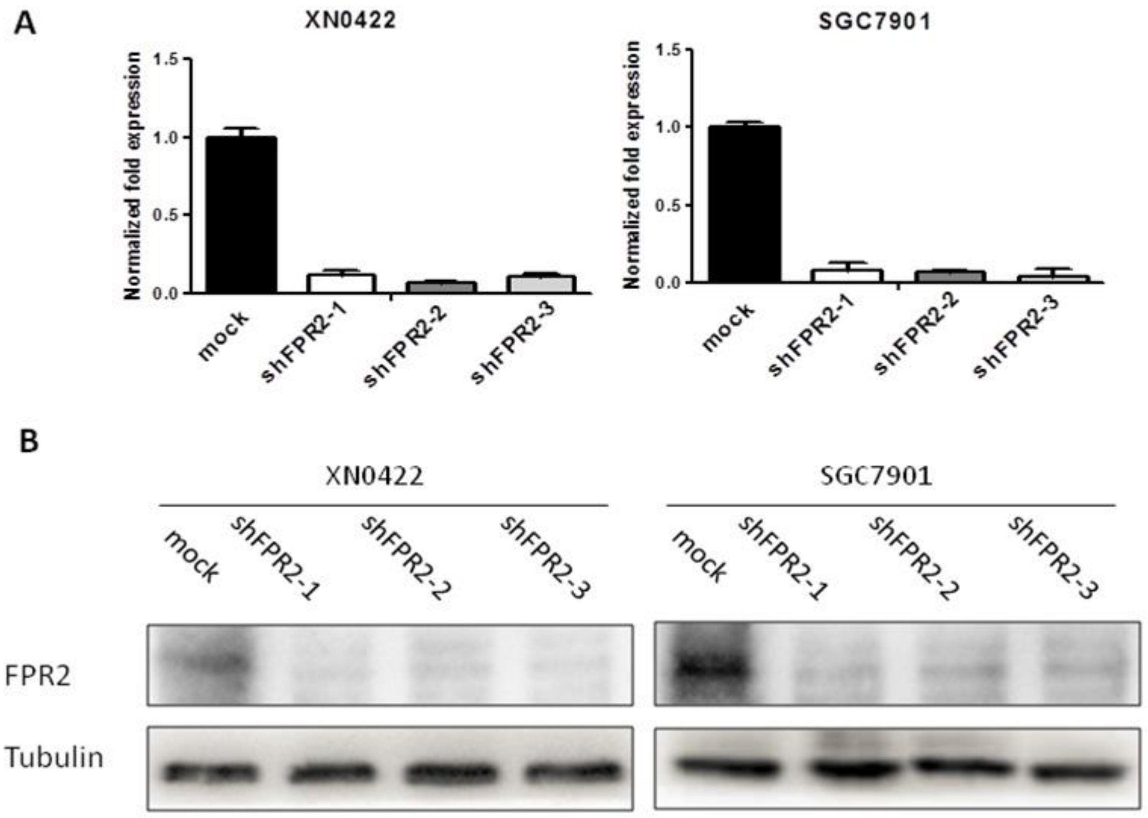

Figure S2. The efficiency of FPR2 knockdown in SGC7901 and XN0422 cells. A, Real-time PCR detected the expression of FPR2 at mRNA level normalized against GAPDH. B, Western blot detected the expression of FPR2 at protein level.

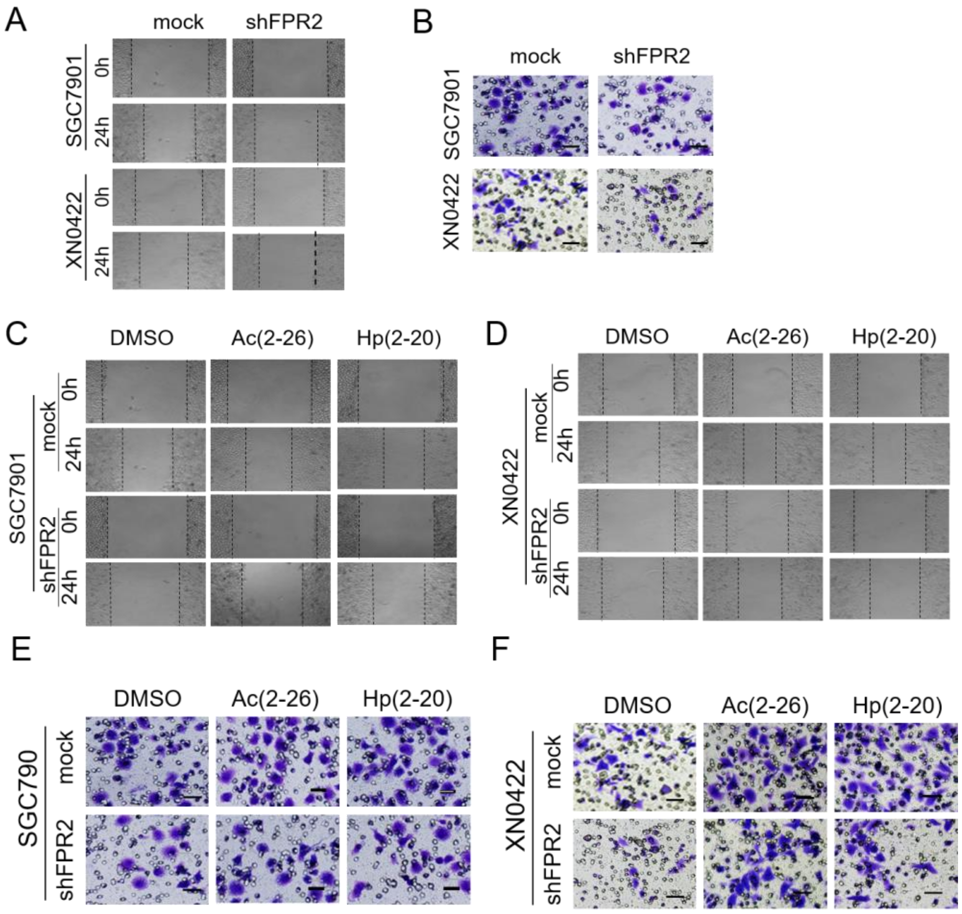

Figure S3. The representative images showing migration and invasion capabilities of XN0422 and SGC7901 cells *in vitro.* A. representative images of wound healing assay for XN0422 and SGC7901 cells with or without FPR2 knockdown. B. representative images of invasion assay for XN0422 and SGC7901 cells with or without FPR2 knockdown. C and D. representative images of wound healing assay showed that stimulation with Hp(2-20) and Ac(2-26) promoted migration of both XN0422 and SGC7901 cells, and this effect was significantly impaired by FPR2-knockdown. E and F. representative images of invasion assay showed that stimulation with Hp(2-20) and Ac(2-26) promoted invasion of both XN0422 and SGC7901 cells, and this effect was significantly attenuated by FPR2-knockdown XN0422 and SGC7901 cells with or without FPR2 knockdown.

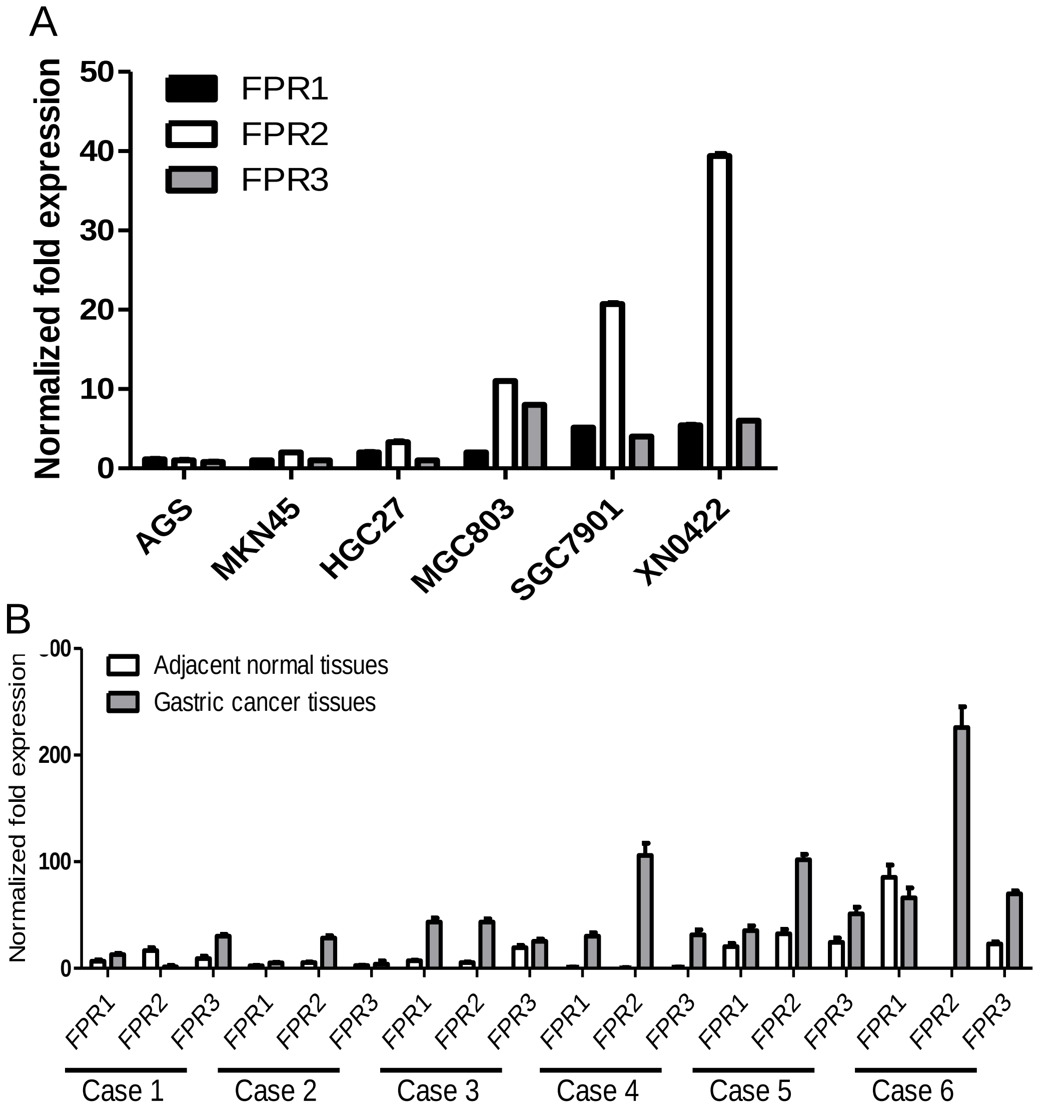

Figure S4. The expression of FPRs in human GC cell lines and cancerous and paired adjacent normal tissues. A. the mRNA expression of FPRs in GC cell lines and primary GC cells detected by qRT-PCR and normalized against GAPDH. B. the mRNA expression of FPRs in fresh GC specimens and their adjacent normal tissues detected by qRT-PCR and normalized against GAPDH.

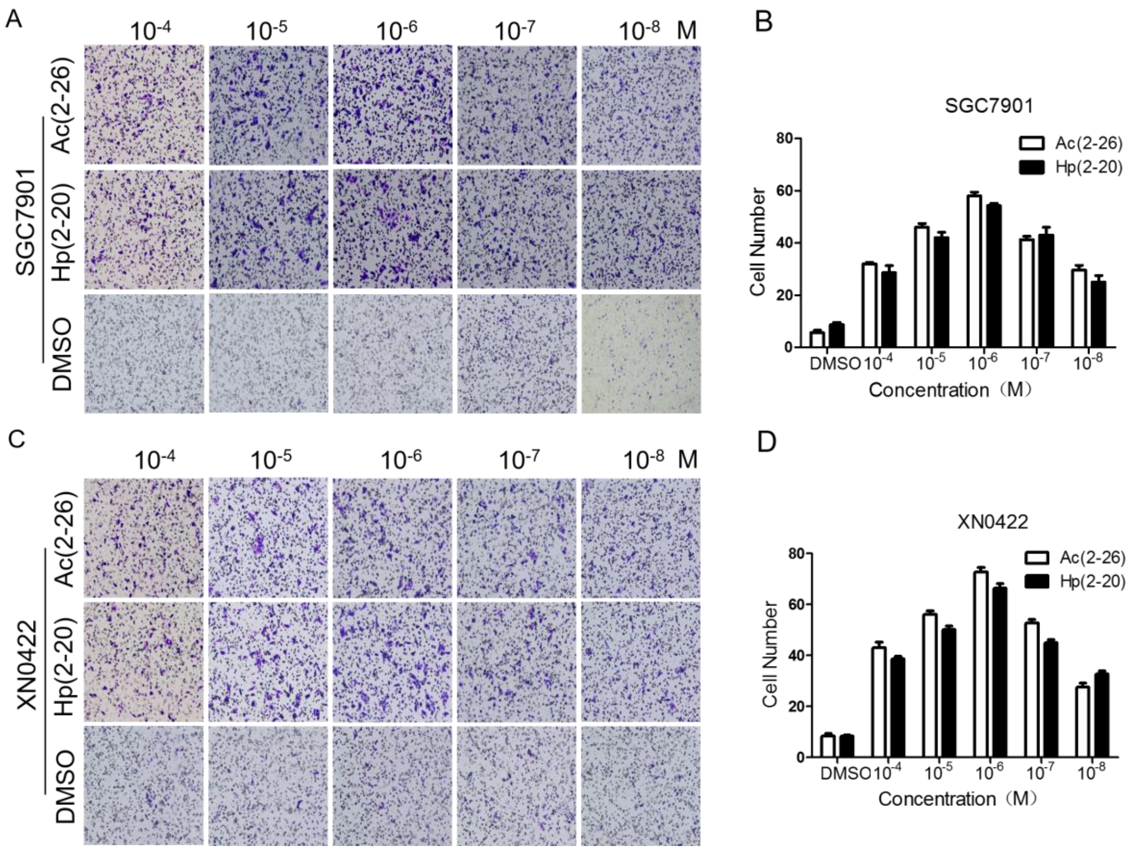

Figure S5. Biological activity analysis of Hp(2–20) and Ac(2-26) by chemotaxis assay.

Transwell chambers (8 μm pore size, Millipore) without matrigel coating were used. The upper wells of the chamber were added with 5×10^4 cells suspended in 200 μL serum-free RPMI-1640 medium. Lower wells of the chamber were added with 600 μL serum-free medium containing different concentrations of Hp(2-20) or Ac(2-26). After incubation for 6 hours at 37 ^{o}C, the chemotactic cells on the lower surface of the membrane were stained and counted. A and B, representative images of SGC7901 cell chemotaxis response to different concentrations of Hp(2-20) or Ac(2-26) and the statistical graph. C and D, representative images of XN0422 cell chemotaxis response to different concentrations of Hp(2-20) or Ac(2-26) and the statistical graph.
